# Changes in epidemiology and antibiotic resistance patterns isolated from paediatric urinary tract infections: a multicenter study in China pre-, during and post-COVID-19

**DOI:** 10.3389/fcimb.2026.1923862

**Published:** 2026-10-19

**Authors:** Liyan Tang, Renyi Tian, Xie Wei, Xiaoyan Yu, Zhongzheng Xiong, Yupei Xiang, Fang Liu, Chunmei Jing

**Affiliations:** 1 Department of Clinical Laboratory Children’s Hospital of Chongqing Medical University, National Clinical Research Center for Children and Adolescents' Health and Diseases, Ministry of Education Key Laboratory of Child Development and Disorders, Chongqing Key Laboratory of Child Rare Diseases in Infection and Immunity, Chongqing, China; 2 Department of Clinical Laboratory, Dian Jiang Peoples Hospital of Chongqing, Chongqing, China; 3 Department of Clinical Laboratory, Chongqing Jiulongpo District Science City People's Hospital, Chongqing, China; 4 Department of Clinical Laboratory, Chongqing Red Cross Hospital (People’s Hospital of Jiangbei District), Chongqing, China

**Keywords:** antibiotic resistance, COVID-19, epidemiology, pediatric, urinary tract infections

## Abstract

**Objective:**

The aim of this study was to determine the epidemiological characteristics and antibiotic resistance patterns of urinary tract infections in children in Chongqing area and to analyze the impact of the COVID-19 pandemic.

**Methods:**

A multicenter retrospective surveillance study was carried out from 2019 to 2024 at four hospitals of the Southwest China Pediatric Laboratory Specialty Alliance. The results were interpreted by the breakpoint criteria recommended by the Clinical and Laboratory Standards Institute (CLSI) M100-S34 guidelines from 2024.

**Results:**

A total of 5618 urine isolates included Gram-negative (71.0%) and Gram-positive bacteria (29.0%) in 53.9% of male and 46.1% of female samples. *E. coli* was the most frequently detected pathogen in children of all ages and gender and levels were greatest for the 4 to 6 year age group (45.7%). Additionally, gender (p = 0.4083) had no significant association with the type of bacterial pathogen isolated, but the six most prevalent isolates grouped by gender displayed significant differences. Resistance of *E. coli* to sulbactam/ampicillin, amikacin, gentamicin and nitrofurantoin displayed a downward trend with time. The average resistance of *E. coli* to carbapenems was < 6% and resistance levels trended upward with time. In contrast, the average resistance of *K. pneumoniae* to carbapenems was >12% and decreased with time while resistance to amikacin, ciprofloxacin, levofloxacin and nitrofurantoin significantly increased with time.Compared to the pre-COVID-19 pandemic, detection of *E.coli, E. faecium* and *K. pneumoniae* decreased post-COVID-19. Post-COVID-19, resistance of *E. coli* and *K. pneumoniae* to piperacillin/tazobactam, cefepime, ciprofloxacin and levofloxacin increased. Resistance of *E. faecium* and *E. faecalis* to penicillin increased significantly as well, while to trimethoprim/sulphamethoxazole decreased markedly (*P* < 0.05).

**Conclusions:**

The COVID-19 pandemic process has brought about changes in pediatric urinary tract infections, emphasizing the necessity of continued monitoring. We found that carbapenems, vancomycin and linezolid remained effective. Age-stratified strategies are essential to manage urinary tract infections in children.The MICs of vancomycin did not drift, but the MICs of linezolid drifted in *E. faecium* and *E. faecalis*. The decreased MICs of *E. faecalis* to linezolid was more obvious and the trend began to emerge in 2022.

## Introduction

Urinary tract infections (UTIs) are the source of inflammatory reactions caused by urinary tract invasion of pathogenic microorganisms. These are some of the most common bacterial infections among children and constitute a frequent reason for hospital admission, medical evaluation and antibiotic prescription (Simões et al., 2020; Autore et al., 2022; Maringhini et al., 2024; Stein Yeshurun et al., 2026). Moreover, as antibiotic resistance in bacterial pathogens rises, the situation becomes increasingly complex (Afsharipoor et al., 2025). For instance, the untimely treatment of UTIs may lead to renal scarring, hypertension, chronic kidney disease or even severe systemic infection (sepsis) or local infection (perirenal abscess) (Lungu et al., 2024; Barola et al., 2025; Moreland et al., 2025). These complications are especially severe in newborns and infants. The global prevalence of UTIs in children is ~8% and overall, in adults ranges from 3-5% in female and 1% in male patients (Daniel et al., 2023; Barola et al., 2025; Kılıç and Küçükkelepçe, 2025). UTIs affect 150 million people per year worldwide (Flores-Mireles et al., 2015).

The key to UTI treatment is to promptly identify the responsible pathogen and precisely prescribe medication based on the results of drug sensitivity tests. Worldwide, the antibiotic resistance of pathogenic bacteria causing UTIs is currently ascendant although drug resistance may vary by region, patient age and time period. Therefore, understanding the pathogens and their antibiotic resistance patterns in a particular region is of vital importance for individualized and precise clinical treatment (Mirsoleymani et al., 2014). Towards these ends, urine culture is the gold standard diagnostic method for UTIs (Lopes Araújo-Filho et al., 2023; Belko and Pohl, 2024) and is used to accurately identify and monitor drug resistance of the pathogens and concurrently provides a basis for guiding clinical medication. Since children have immature immunity, their treatment becomes problematic since some antibacterials are restricted for use in these patients. Therefore, the current situation of antibacterial drug resistance in children differs from that in adults and requires additional attention.

Severe Acute Respiratory Syndrome Coronavirus 2 (SARS COV-2), a single-stranded RNA virus with positive polarity that caused Coronavirus disease (COVID), was discovered for the first time in November 2019 in Wuhan, China. It has influenced all of humanity since that day and has resulted in a complete change in our daily routine. Due to the adoption of protective measures such as wearing masks, social distancing and strict adherence to hygiene rules, the incidence of some diseases transmitted through contact (such as upper respiratory tract infections and asthma) has decreased (Hoberman et al., 1999; Conway et al., 2007). In contrast, there are relatively few studies on the occurrence and prevalence of UTIs in children during this pandemic. There are currently no multicenter studies planned either in China or abroad on the impact of COVID-19 on the epidemiology and antibiotic resistance patterns in children UTIs. To remedy this situation, the current study investigated the epidemiological characteristics and antibiotic resistance patterns of UTIs in children in the Chongqing area from 2019 to 2024 and analyzed the impact of COVID-19 on these infections. The goal was to provide a reference for the early diagnosis and empirical medicinal treatment of UTIs in children. This is also of great value for preventing and controlling nosocomial infections and curbing the spread of drug resistance.

## Materials and methods

### Patients and enrollment of bacteria

Between January 2019 and December 2024, four hospitals of the Southwest China Pediatric Laboratory Specialty Alliance took part in this study. Patients included were children aged <18 years with community-acquired UTI who presented with a single UTI episode in the study. Patients aged ≥18 years, those with two or more UTIs episodes, underlying congenital anomalies of the kidney and urinary tract (CAKUT), duplicate isolates, and bacterial colonization without clinical evidence of infection were excluded from the study.All medical institutions involved in the multicenter study had adopted the unified inclusion and exclusion criteria.

### Urine samples culture

Mid-stream or catheterized urine samples were collected in a sterile container. According to the guideline of the American Academy of Pediatrics, pyuria and presence of ≥ 5×10^4^ colonies in a catheterized urine samples were accepted as urinary infection (Reaffirmation of AAP clinical practice guideline: The diagnosis and management of the initial urinary tract infection in febrile infants and young children 2-24 months of age, 2016). According to the updated Italian recommendations for the diagnosis and treatment of urinary tract infection in children, the presence of ≥ 5×10^4^ colonies in mid-stream urine samples was accepted as urinary infection (Ammenti et al., 2020).

### Isolation and identification of strains

Bacterial isolates from agar plate culture were identified using a variety of methods that included Matrix-assisted laser desorption/ionization time-of-flight mass spectrometry, the VITEK2-Compact automatic bacterial identification and drug sensitivity instrument (bio Mérieux, France) and the BD Phoenix automatic microbiological identification/drug sensitivity system (Beckton Dickenson, Sparks, MD, USA).

### Antibiotic sensitivity testing

Antimicrobial susceptibility testing (AST) was performed using automated systems that included the VITEK2 Compact (bio Mérieux) or the BD Phoenix (Becton Dickinson). AST results were interpreted according to Clinical and Laboratory Standards Institute (CLSI) M100-S34 guidelines (2024) (Clinical and Laboratory Standards Institute (CLSI), 2024). The control strains for these procedures were *Pseudomonas aeruginosa* (ATCC 27853), *Escherichia coli* (ATCC 25922), *Klebsiella Pneumoniae* (ATCC 700603) and *Enterococcus faecalis* (ATCC 29212) (American Type Culture Collection, Manassas, VA, USA).

### Definitions

The study period (2019--2024) was divided into the pre-COVID-19 (2019), COVID-19 pandemic (2020–2022) and post-COVID-19 pandemic (2023–2024).

Age stratification was defined as ≤1 year, 2-3 years, 4-6 years and ≥7 years, corresponding to infancy, toilet-training period, pre-school age and school-age. These developmental milestones are associated with distinct UTI clinical manifestations, gender distribution and voiding-related risk factors, and similar age-cut-off strategies have been adopted in previous pediatric UTI retrospective epidemiological studies (Daniel et al., 2023; Isac et al., 2024).

### Statistical analysis

Antimicrobial resistance data were analyzed using WHONET 5.6 software to obtain the MIC_50_ and MIC_90_ values. Prism 8 (GraphPad, Boston, MA, USA) was used for statistical visualization. The temporal trends, age distributions and detection levels of bacterial resistance phenotypes were analyzed using Fisher’s exact test or the Chi-square test. To evaluate linear trends in detection rates and MIC values for the same drug across different years and groups, the Chi-square test for trend was applied. Dynamic changes in MIC values over time were assessed using the non-parametric Spearman correlation test. Statistical significance was established at a two-tailed P-value < 0.05.

## Results

### Distribution of clinical isolates in paediatric UTIs

From 2019 to 2024, a total of 5618 pathogenic bacteria were isolated from positive urine samples, including 3988 (71.0%) Gram-negative and 1630 (29.0%) Gram-positive isolates. In total, 100 distinct uropathogenic species were identified, consisting of 66 Gram-negative and 34 Gram-positive species. *E. coli* was the most predominant Gram-negative pathogen, accounting for 37.3% (2093/5618) of all isolates. Other frequently recovered pathogens comprised *E. faecium* (12.9%), *E. faecalis* (9.0%), *K. pneumoniae* (9.0%), *Proteus mirabilis* (5.1%), *P. aeruginosa* (3.5%), *Enterobacter cloacae* (2.1%), *Morganella morganii* (1.8%), *Proteus vulgaris* (1.8%), and *Klebsiella oxytoca* (**Figure 1a**). The peak detection rates of *E. coli*, *E. faecium*, and *K. pneumoniae* occurred in 2019, 2020, and 2024, respectively, and the prevalence of these three pathogens declined in the post-COVID-19 period (**Figures 1b, c**).

**Figure 1:**
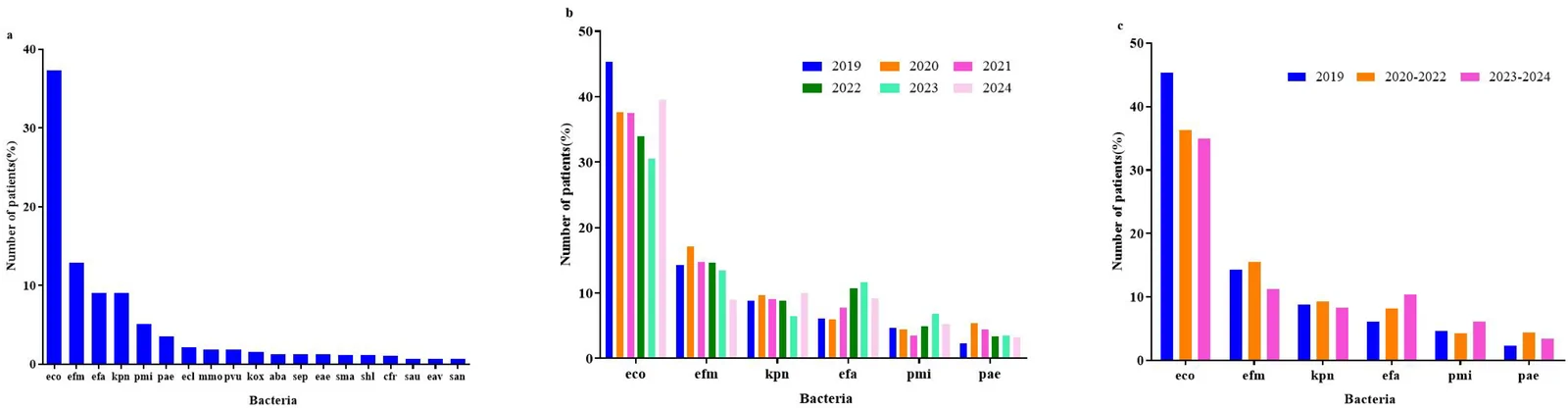
Distributions of bacterial species identified urine cultures from 2019 to 2024 used in this study. **(a)** The 20 most prevalent bacterial types identified in this study as paediatric UTIs. Relative proportions represented by the 6 most prevalent bacteria in paediatric UTIs grouped by **(b)** year and **(c)** pre-, during and post-COVID-19 periods. eco, *E. coli*; efm*. E. faecium*; efa, *E. faecalis*; kpn, *K. pneumoniae*; pmi, *P. mirabilis*; pae, *P. aeruginosa*; ecl, *Enterobacter cloacae*; mmo, *Morganella morganii*; pvu, *Proteus vulgaris*; kox, *Klebsiella oxytoca*; aba, *Acinetobacter baumannii*; sep, *Staphylococcus epidermidis*; eae, *Enterobacter aerogenes*; sma, *Serratia marcescens*; shl, *Staphylococcus haemolyticus*; cfr, *Citrobacter freundii*; sau, *Staphylococcus aureus*; eav, *Enterococcus avium*; san, *Streptococcus anginosus.*.

### Distribution of gender and age groups in paediatric UTIs

Our data showed that pediatric UTI isolates were obtained from 53.9% (3026/5618) male and 46.1% (2592/5618) female patients. Males were most susceptible at age ≤1 year, whereas females had the highest susceptibility at ≥7 years. Higher prevalence among males was observed in the ≤1-year (59.8% vs 40.2% female) and 2-3-year age groups (54.7% vs 45.3% female); by contrast, females predominated in the 4-6-year and ≥7-year subgroups (**Figure 2a**). *E. coli* was the leading pathogen across all age strata: 45.7% in 4-6 years, 42.2% in 2-3 years, 34.8% in ≥7 years, and 32.8% in ≤1 year. The second most common isolates per age group were *E. faecium* (22.5%) for ≤1 year, *E. faecalis* (9.9%) for 2-3 years, *K. pneumoniae* (8.1%) for 4-6 years, and *E. faecalis* (11.8%) for ≥7 years (**Figure 2b**). Stratified by sex, the predominant pathogens in males were *E. coli* (30.4%), *E. faecalis* (12.6%), *E. faecium* (10.2%), *K. pneumoniae* (8.7%), *P. mirabilis* (5.8%), and *P. aeruginosa* (4.2%). In females, the top pathogens were *E. coli* (45.1%), *E. faecium* (16.2%), *K. pneumoniae* (9.1%), *E. faecalis* (4.7%), *P. mirabilis* (4.3%), and *P. aeruginosa* (2.6%) (**Figure 2c**). Overall, gender was not significantly associated with pathogen species (p = 0.4083), yet significant inter-gender differences existed among the six most frequently isolated species.

**Figure 2:**
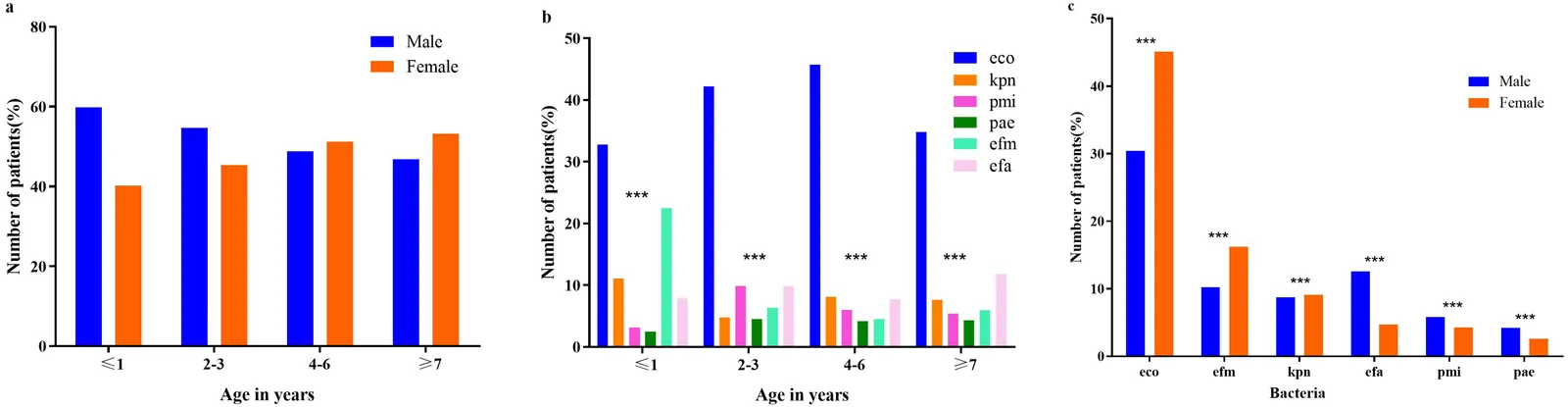
Distribution of gender and age groups for urinary tract infection patients. **(a)** Distribution of male and females among different age groups. **(b)** The six most prevalent bacteria among different age groups. **(c)** The six most prevalent bacterial species grouped by sex. eco, *E. coli*; efm*. E. faecium*; efa, *E. faecalis*; kpn, *K. pneumoniae*; pmi, *P. mirabilis*; pae, *P. aeruginosa.* ***P < 0.001.

### The prevalence of antibiotic resistance stratified by age

Resistance rates to ciprofloxacin and levofloxacin differed significantly across four age groups for *E. coli* and *K. pneumoniae* (**Figures 3a, b**). Significant age-related differences in ceftazidime resistance were found in *P. mirabilis* and *P. aeruginosa* (**Figures 3c, d**), as were levofloxacin resistance rates for *E. faecalis* and *E. faecium* (**Figures 3e, f**).

**Figure 3:**
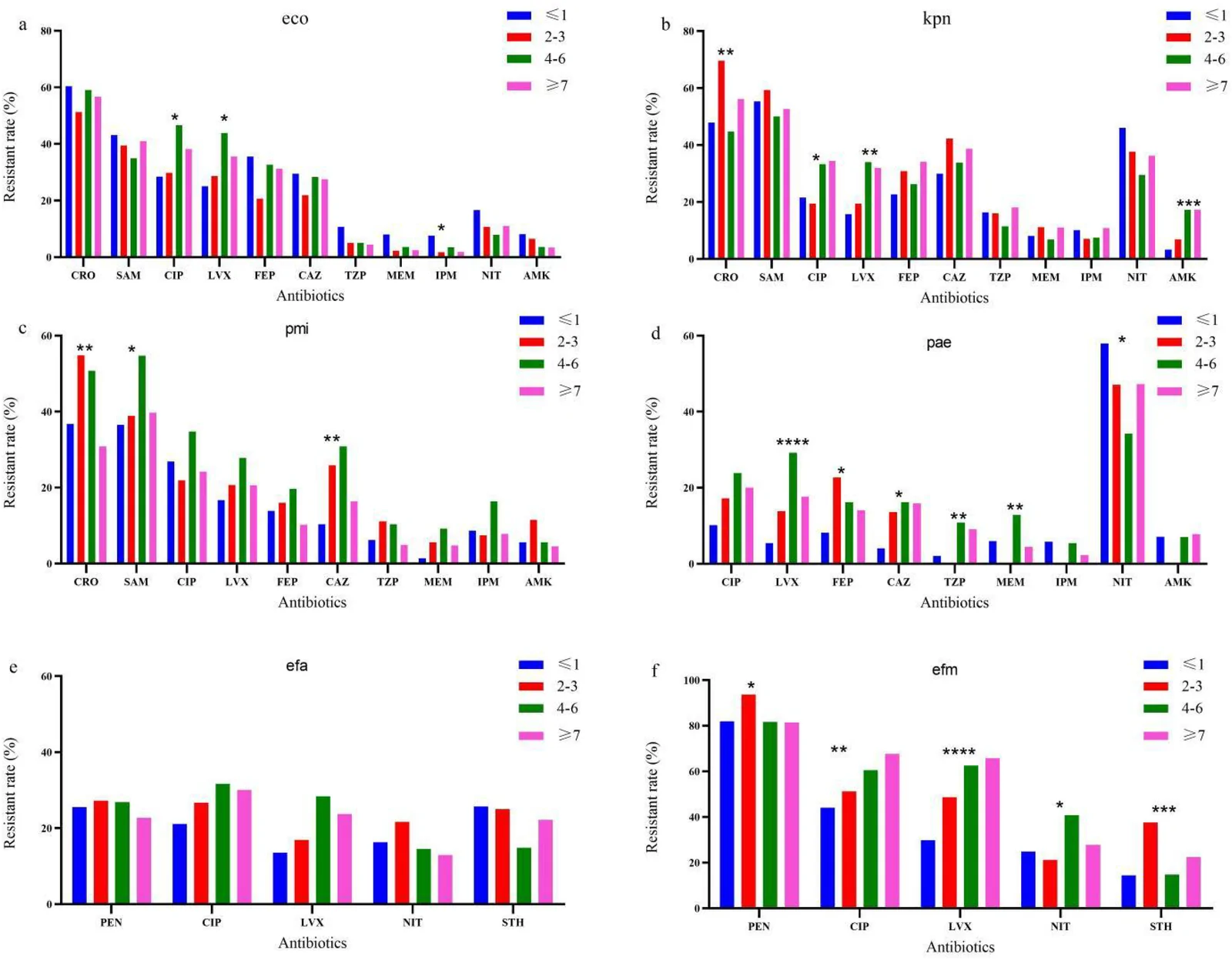
The prevalence of antibiotic resistance stratified by age **(a)** eco. **(b)** kpn. **(c)** pmi. **(d)** pae.**(e)** efa. **(f)** efm. eco, *E. coli*; efm*. E. faecium*; efa, *E. faecalis*; kpn, *K. pneumoniae*; pmi, *P. mirabilis*; pae, *P. aeruginosa.*CRO, ceftriaxone; SAM, ampicillin/sulbactam; CIP, ciprofloxacin; LVX, levofloxacin; GEN, gentamicin; FEP, cefepime; CAZ, ceftazidime; TZP, piperacillin/tazobactam; MEM, meropenem; IPM, imipenem; NIT, nitrofurantoin; AMK, amikacin; PEN, penicillin; SXT, trimethoprim/sulphamethoxazole; STH, high-level streptomycin. *P < 0.05, **P < 0.01, ***P < 0.001.

### Antimicrobial resistance of *E. coli* and *K. pneumoniae*

From 2019 to 2024, resistance of *E. coli* to sulbactam/ampicillin, amikacin, gentamicin and nitrofurantoin displayed a downward trend. In particular, sulbactam/ampicillin decreased from 57.9% in 2019 to 36.5% in 2024 and this was a significant change (*P* < 0.001). The average resistance of *E. coli* to cephalosporins was < 65% and to carbapenems was < 6%, although the general trend for resistance rose as time progressed (**Figure 4a**). The resistance of *K. pneumoniae* to amikacin, ciprofloxacin, levofloxacin and nitrofurantoin also displayed an obvious upward trend (*P* < 0.05). In particular, average resistance of *K. pneumoniae* to carbapenems was> 12% and this differed from *E. coli* where resistance decreased with time (**Figure 4b**). *E. coli* resistance to ceftriaxone, ciprofloxacin, levofloxacin and gentamicin were greater that for *K. pneumoniae* (*P* < 0.05) while resistance to sulbactam/ampicillin, ceftazidime, piperacillin/tazobactam, imipenem, meropenem and nitrofurantoin were significantly lower (*P* < 0.05) (**Figure 4c**).

**Figure 4:**
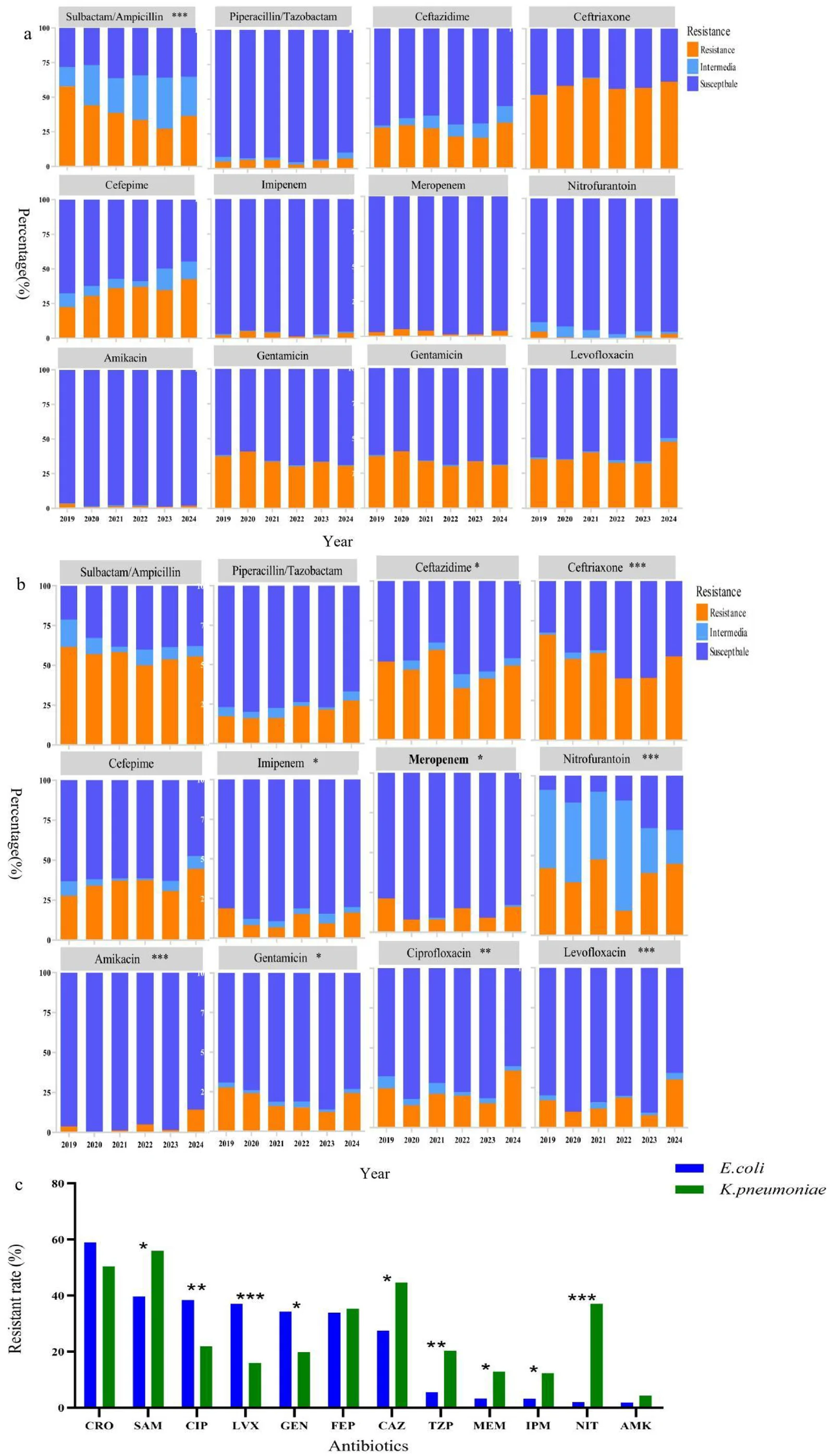
Antimicrobial Resistance of *E. coli and K. pneumoniae.* **(a)** Resistance of *E. coli* to routine antibiotics grouped by year. **(b)** Resistance of *K. pneumoniae* to routine antibiotics grouped by year. **(c)** Difference of resistance for *E. coli and K. pneumoniae.* CRO, ceftriaxone; SAM, ampicillin/sulbactam; CIP, ciprofloxacin; LVX, levofloxacin; GEN, gentamicin; FEP, cefepime; CAZ, ceftazidime; TZP, piperacillin/tazobactam; MEM, meropenem; IPM, imipenem; NIT, nitrofurantoin; AMK, amikacin. *P < 0.05, **P < 0.01, ***P < 0.001.

### Antimicrobial resistance of *E. faecium* and *E. faecalis*

The average resistance rates of *E. faecium* and *E. faecalis* to penicillin were 91.5% and 63.7%, respectively, while their corresponding ciprofloxacin resistance rates were 6.5% and 19.9%. Both enterococcal species exhibited increasing resistance trends over the study period (**Figures 5a, b**). No vancomycin- or linezolid-resistant *E. faecalis* isolates were detected in this cohort. Compared with *E. faecalis*, *E. faecium* showed significantly higher resistance to penicillin, ciprofloxacin, levofloxacin, and nitrofurantoin (all P<0.05). In contrast, *E.faecium* exhibited lower resistance to high-level streptomycin than *E. faecalis* (**Figure 5c**).

**Figure 5:**
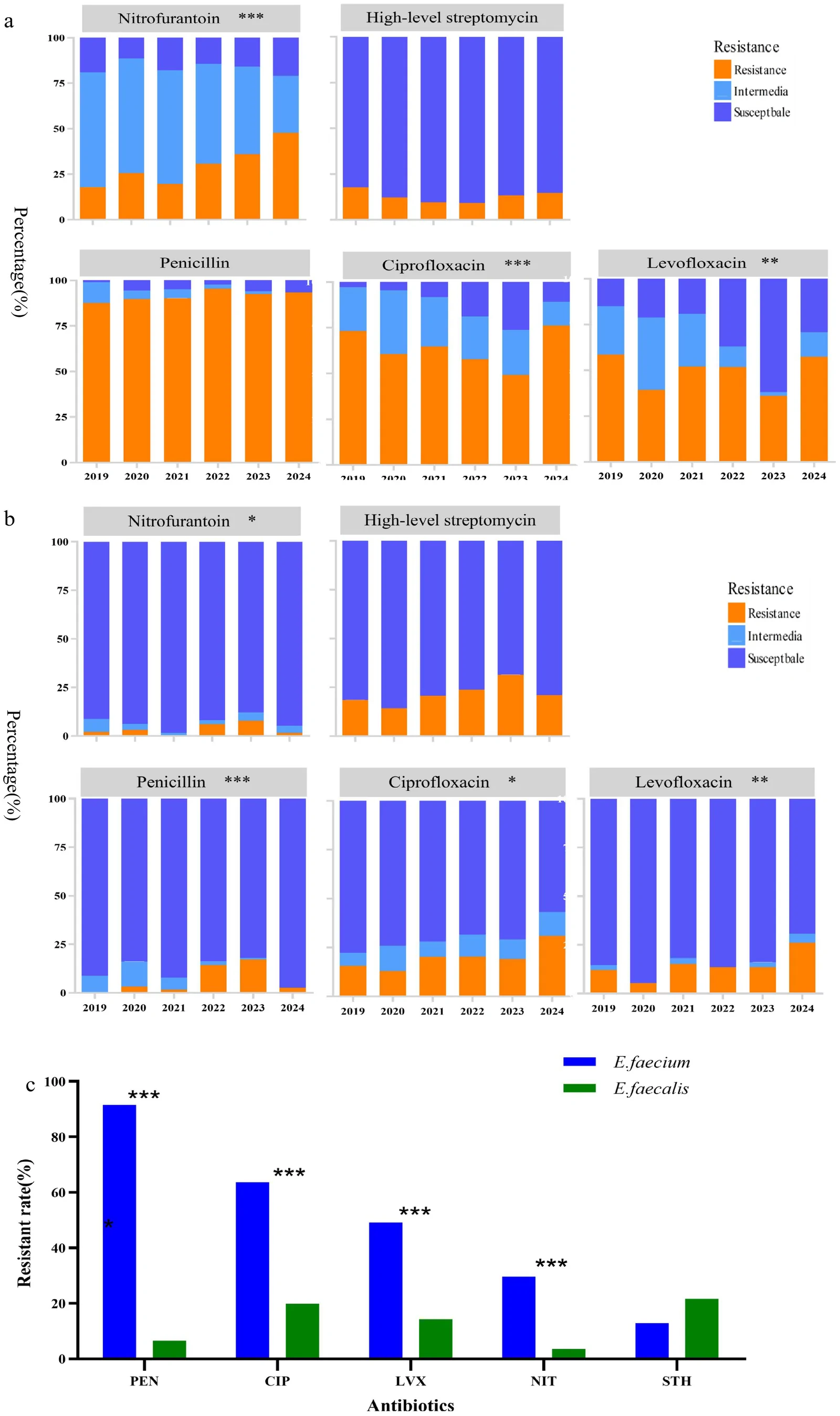
Antimicrobial Resistance of *E. faecium* and *E. faecalis.* **(a)** Resistance of *E. faecium* to routine antibiotics grouped by year. **(b)** Resistance of *E. faecalis* to routine antibiotics by year. **(c)** Difference of resistance between *E. faecium* and *E. faecalis.* PEN, penicillin; CIP, ciprofloxacin; LVX, levofloxacin; NIT, nitrofurantoin; STH, high-level streptomycin. *P < 0.05, **P < 0.01, ***P < 0.001.

### ESBL-producing and carbapenem resistant-*E. coli* and *K. pneumoniae*

We further analyzed the temporal trends of extended-spectrum β-lactamase (ESBL) production and carbapenem resistance in *E. coli* and *K. pneumoniae*. The prevalence of ESBL-producing *E. coli* increased gradually from 54.0% in 2019 to 62.2% in 2024, whereas the proportion of ESBL-producing *K. pneumoniae* decreased significantly from 66.2% to 52.7% over the same period (P < 0.05) (**Figure 6a**). Similarly, carbapenem-resistant *E. coli* showed an upward trend, rising from 2.7% (2019) to 4.9% (2024). In contrast, carbapenem resistance in *K. pneumoniae* declined significantly from 23.1% (2019) to 18.9% (2024) (P < 0.05). Notably, carbapenem-resistant *K. pneumoniae* reached its peak prevalence of 23.1% in 2019 and a minimum of 8.0% in 2020 (**Figure 6b**).

**Figure 6:**
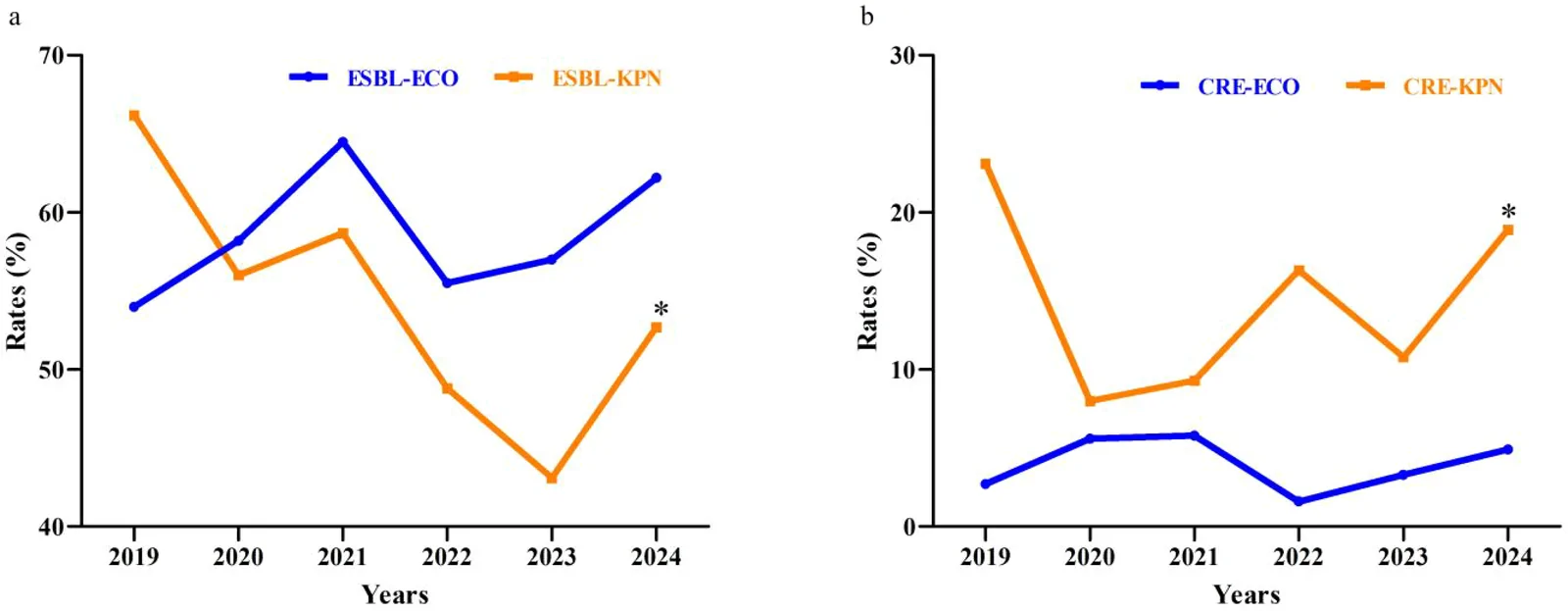
Levels of ESBL-producing and Carbapenem resistant *E. coli* and *K. pneumoniae* from 2019 to 2024. **(a)** ESBL-producing *E. coli* and *K. pneumoniae*. **(b)** Carbapenem resistant for *E. coli* and *K. pneumoniae*. ESBL-ECO, ESBL-producing *E. coli*; ESBL-KPN, ESBL-producing *K. pneumoniae*; CRE-ECO, carbapenem resistant-*E.coli*; CRE-KPN, carbapenem resistant-*K. pneumoniae*. *P < 0.05.

### Resistance of ESBL-producing and carbapenem resistant-*E. coli* and *K. pneumoniae*

Compared with non-ESBL-producing *E. coli* and *K. pneumoniae*, ESBL-producing strains of both species showed significantly elevated resistance to ampicillin/sulbactam, cefepime, imipenem, meropenem, ciprofloxacin, levofloxacin, and gentamicin across the study period (P < 0.05) (**Figures 7a, b**). Additionally, carbapenem-resistant E. coli and K. pneumoniae exhibited broad resistance to all tested antimicrobial agents (**Figures 7c, d**).

**Figure 7:**
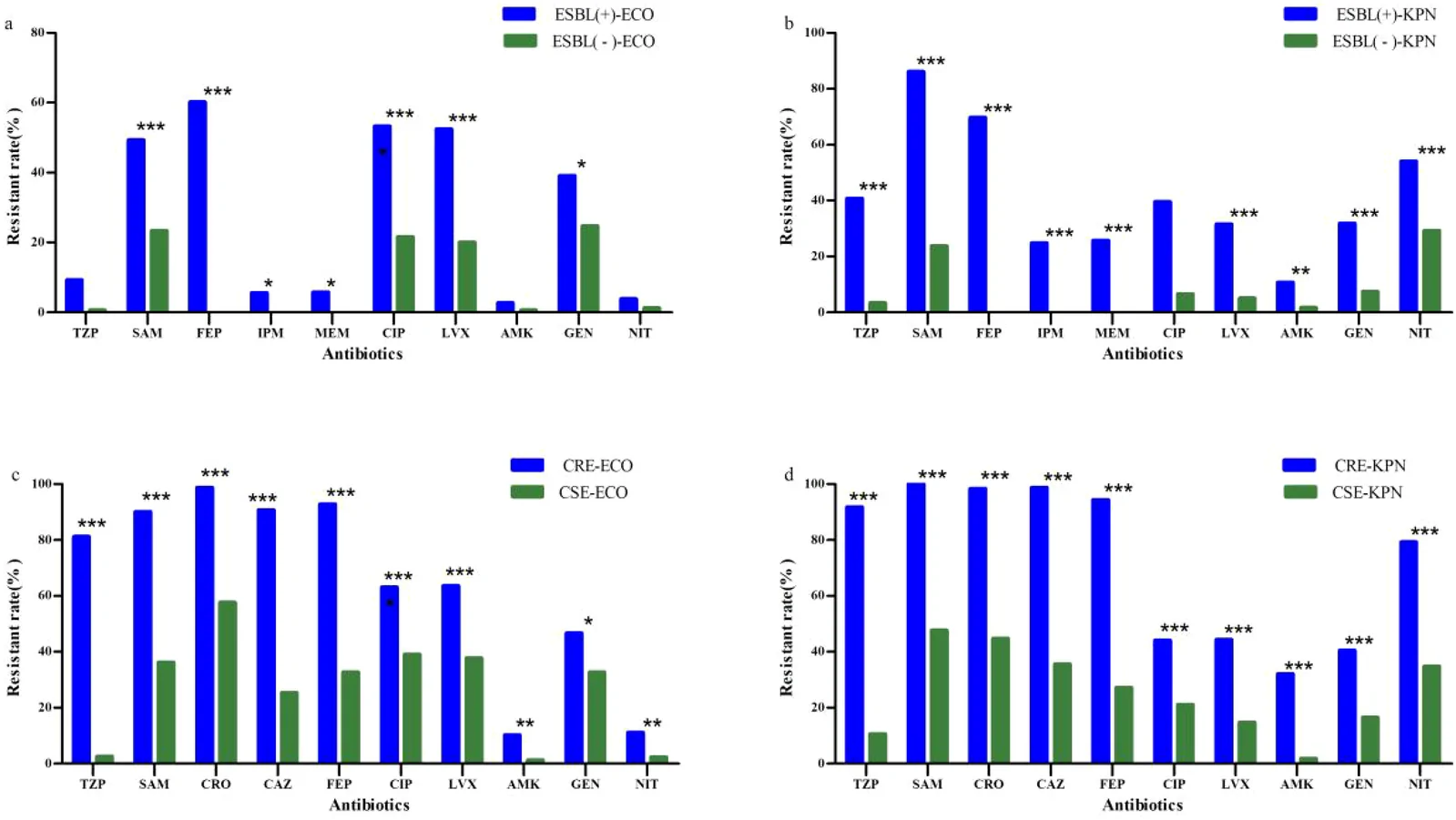
Resistance of ESBL-producing and Carbapenem resistant-*E. coli* and *K. pneumoniae.* **(a)** Resistance of ESBL(+)-ECO and ESBL(-)-ECO. **(b)** Resistance of ESBL(+)-KPN and ESBL(-)-KPN. **(c)** Resistance of CRE-ECO and CSE-ECO. **(d)** Resistance of CRE-KPN and CSE-KPN. ESBL(+)-ECO, ESBL-producing *E. coli*; ESBL(-)-ECO, non ESBL-producing *E. coli*; ESBL(+)-KPN, ESBL-producing *K. pneumoniae*; ESBL(-)-KPN, non ESBL-producing *K. pneumoniae*; CRE-ECO, carbapenem resistant-*E.coli*; CSE-ECO, carbapenem sensitive-*E.coli*; CRE-KPN, carbapenem resistant-*K. pneumoniae;* CSE-KPN, carbapenem sensitive-*K. pneumoniae.* *P < 0.05, **P < 0.01, ***P < 0.001.

### The impact of COVID-19 pandemic on the drug resistance rate of UTI pathogenic bacteria in children

Further temporal comparison revealed distinct post-pandemic resistance shifts relative to the pre-COVID-19 era. Both *E. coli* and *K. pneumoniae* displayed increased post-pandemic resistance to piperacillin/tazobactam, cefepime, ciprofloxacin, and levofloxacin. Conversely, *E. coli* resistance to ampicillin/sulbactam decreased significantly from 57.9% to 31.9% (P < 0.001), and *K. pneumoniae* resistance to ceftriaxone declined substantially from 66.2% to 49.8% (P < 0.05). For amikacin and nitrofurantoin, *K. pneumoniae* resistance showed an upward trend, whereas *E. coli* resistance trended downward. Regarding enterococci, post-pandemic *E. faecium* and *E. faecalis* demonstrated significantly higher penicillin resistance (P < 0.05) (**Figure 8**).

**Figure 8:**
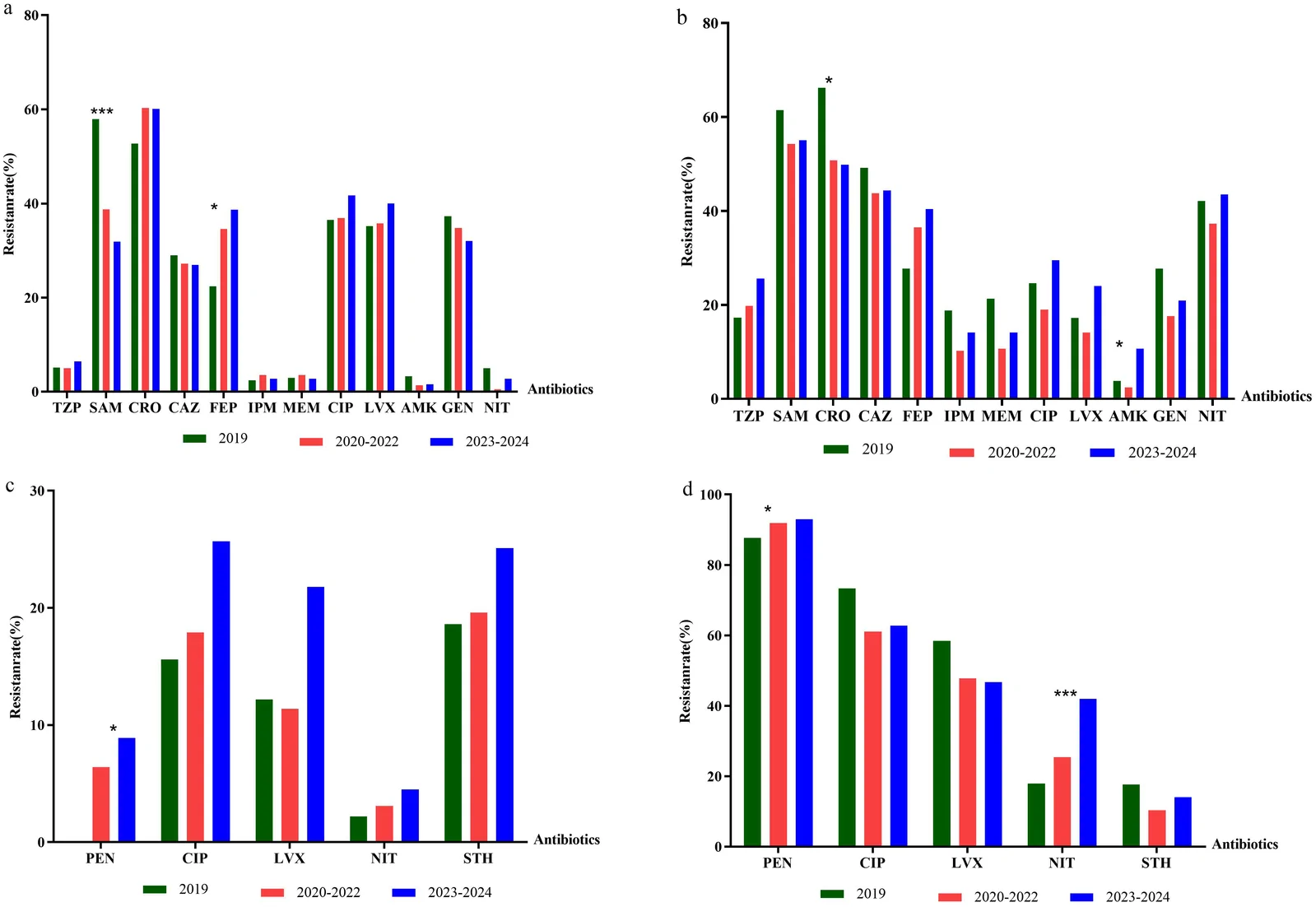
Antibiotic resistance to the indicated study groups pre-, during and post-COVID-19. Prevalence of antibiotic resistance for **(a)** *E. coli*, **(b)** *K. pneumoniae*, **(c)** *E. faecalis* and **(d)** *E. faecium.* CRO, ceftriaxone; SAM, ampicillin/sulbactam; CIP, ciprofloxacin; LVX, levofloxacin; GEN, gentamicin; FEP, cefepime; CAZ, ceftazidime; TZP, piperacillin/tazobactam; MEM, meropenem; IPM, imipenem; NIT, nitrofurantoin; AMK, amikacin. PEN, penicillin; SXT, trimethoprim/sulphamethoxazole; STH, high-level streptomycin. *P < 0.05, ***P < 0.001.

### MIC drift of vancomycin and linezolid against *E. faecium* and *E. faecalis*

*E. faecalis* exhibited a significantly higher proportion of vancomycin MICs ≥ 2 μg/mL than *E. faecium* (17.7% [90/509] vs. 5.0% [37/735]). In contrast, the prevalence of linezolid MICs ≥ 2 μg/mL was significantly lower in *E. faecalis* than in *E. faecium* (69.0% [351/509] vs. 82.0% [603/735], P < 0.05). Both *E. faecium* and *E. faecalis* showed a significant decreasing trend in the prevalence of linezolid MICs ≥ 2 μg/mL over time (P < 0.001). Additionally, the vancomycin MIC values for *E. faecalis* were comparable to or higher than those for *E. faecium* (**Table 1**).

**Table 1:** MIC distribution patterns of vancomycin and linezolid resistance in *E. faecium* and *E. faecalis.*.

Year	Strains	Vancomycin							Linezolid					
		MIC distribution (ug/mL)				Proportion of MIC ≥2 (%)	MIC_50_ (ug/mL)	MIC_90_ (ug/mL)	MIC distribution (ug/mL)			Proportion of MIC ≥2 (%)	MIC_50_ (ug/mL)	MIC_90_ (ug/mL)
		0.5	1	2	4				1	2	4			
2019														
*E. faecalis*	45	1	36	8	0	17.8	1	2	3	42	0	93.3	2	2
*E. faecium*	106	48	51	6	1	6.6	0.5	1	9	97	0	91.5	2	2
2020														
*E. faecalis*	31	3	22	6	0	19.4	1	2	1	25	5	96.8	2	4
*E. faecium*	89	28	59	1	1	2.2	1	1	5	82	2	94.4	2	2
2021														
*E. faecalis*	64	7	44	13	0	20.3	1	2	2	62	0	96.9	2	2
*E. faecium*	122	24	95	2	1	2.5	1	1	11	110	1	91.0	2	2
2022														
*E. faecalis*	98	3	77	17	1	18.4	1	2	20	78	0	79.6	2	2
*E. faecium*	133	27	97	9	0	6.8	1	1	26	107	0	80.5	2	2
2023														
*E. faecalis*	116	9	85	19	3	19.0	1	2	46	70	0	60.3	2	2
*E. faecium*	134	61	64	4	5	6.7	1	1	25	109	0	81.3	2	2
2024														
*E. faecalis*	155	5	127	11	12	14.8	1	2	86	67	2	44.5	2	2
*E. faecium*	151	100	44	6	1	4.6	0.5	1	56	95	0	62.9	2	2

MIC, minimum inhibitory concentration,; MIC_50_, minimum inhibitory concentration inhibiting 50% of isolates; MIC_90_, minimum inhibitory concentration inhibiting 90% of isolates.

## Discussion

Our multicenter study confirmed that the COVID-19 pandemic had changed bacterial profiles and antibiotic resistance patterns in pediatric UTIs. As far as we know, this is the first multicenter study on COVID-19 pandemic influence to epidemiology and antibiotic resistance patterns in pediatric UTIs conducted in China, offering essential insights for optimizing antibiotic treatment strategies in pediatric UTI.

Our study indicated that most organisms detected were Gram-negative (71.0%) and this was less than reported in other studies at 89.4% (Cag et al., 2021) and 88.5% (Bozo et al., 2025). The six most prevalent bacterial species we identified were *E. coli, E. faecium*, *K. pneumoniae, E. faecalis*, *P. mirabilis* and *P. aeruginosa*, that were consistent with reports from Turkey and Yakup (Cag et al., 2021; Samanci and Pınarbaşı, 2023). UTI infections caused by *E. coli* typically range from 60 to 90%. For instance, recent studies reported levels of 80-90% (Tullus and Shaikh, 2020), 63.7% (Afsharipoor et al., 2025), 87.0% (Bozo et al., 2025) and 70.8% (Whelan et al., 2024). In our study, *E. coli* levels were at 37.3%, which was comparable to the 38.84% reported by Raluca Isac (Isac et al., 2021). Nonetheless, all these studies indicated that *E. coli* was the most prevalent UTI pathogen in children. In addition, we identified *K. pneumoniae* as a primary UTI pathogen in children and this has been previously reported for children (Lutter et al., 2005). A recent study in Iran (Mirsoleymani et al., 2014) indicated levels of 36.2% of all UTIs in children. In contrast, a Turkish study found that *Proteus* spp. was the second most common bacterium causing pediatric UTIs (19.0%) although *K. pneumoniae* was still highly represented (14.7%) (Erol et al., 2018).

The detection of *E. coli* and *K. pneumoniae* as UTI pathogens also varied by age group and gender. The predominance of male UTIs found in our study (53.9 vs. 46.1% female) was consistent with the findings reported by Raluca Isac (58.07% vs. 41.93% female) (Isac et al., 2021), yet inconsistent with the female-predominant findings reported from Romania (43% vs. 57% female) (Isac et al., 2024). Also, males had a higher prevalence than females in the ≤1 year group (59.8 vs. 40.2% females) and 2–3 years (54.7 vs. 45.3% females) and was consistent with a report that males accounted for 85.3% of infant UTIs (Păcurar et al., 2025). Our results also indicated a predilection for females to be infected more than males in the 4–6 year (48.8 vs. 51.2% females) and ≥7 year (46.8 vs. 53.2% females) groups. This was also consistent with a predilection for girls in the 5–10 year age group to suffer from UTIs (Samanci and Pınarbaşı, 2023). Hence, male predominance is observed in early infancy, especially in uncircumcised males, while UTIs become more common in females as children grow older (Shaikh et al., 2008; Deltourbe et al., 2022; Bagnasco et al., 2025). The possible reason may be the specificity of embryonic development and the delayed maturation of the ureter, boys have a much higher risk of severe congenital urinary tract malformations (obstructive urinary tract malformations) associated with UTIs than girls of the same age (Sato et al., 2023). This would lead to poor urine excretion or reflux that prolongs retention in the urinary tract and significantly increases the risk of infection. The second reason is that most boys have physiological phimosis (Puhl et al., 2022; Moreno et al., 2024) that also provides favorable conditions for bacterial colonization and multiplication near the urethral opening. All males in this study were uncircumcised since circumcision is an extremely rare practice with boys of ≤3 years. Girls > 4 years have a higher risk of UTIs than boys and the most likely reason is that girls have shorter urethras which are closer to the anus, making it easier for pathogens to ascend and cause infections. Therefore, suggests gender- and age-specific factors require further research for future strategies but the general trends are apparent.

Our research results also indicated showed that *E. coli* was the most common pathogen for each age group and this differed from the reports in the Turkey study where non-*E. coli* infections were more common up to 2 years and *E. coli* infections were more common > 2 years (Samanci and Pınarbaşı, 2023). We found that *E. coli* infections were more common in girls (45.1%) and non-*E. coli* infections were more common in boys (30.4%)(p<0.001), consistent with the Turkey results (female 68.1%) (Samanci and Pınarbaşı, 2023). We also found a more prevalent association of *K. pneumoniae* at ≤ 1 year (11.1%). This finding aligns with prior studies showing that Enterobacteriaceae, predominantly *E. coli* and *K. pneumoniae*, are common causative pathogens of infantile UTIs (Wong et al., 2025).A bidirectional association exists between ESBL-producing Enterobacteriaceae urinary tract infection and recurrent UTI. A prior history of recurrent UTI is an independent risk factor for ESBL-UTI, largely attributable to repeated antibiotic exposure facilitating the selection of resistant strains (Flokas et al., 2016). Meanwhile, ESBL positivity may increase the risk of subsequent UTI relapse, particularly in children with underlying urinary tract anomalies such as CAKUT (Flokas et al., 2016; Lu et al., 2022). However, after adjusting for anatomical abnormalities and antibiotic exposure, ESBL status alone may not serve as an independent predictor of recurrence (Lu et al., 2022).

The levels of antibiotic resistance used in empirical treatment for patients diagnosed with UTIs should not exceed 20% (Gupta et al., 2011). The Infectious Diseases Society of America reports that it is crucial to understand the local pathogens of urinary tract infections and their antibiotic resistance levels (Alós et al., 2005). Resistance to the third-generation cephalosporins widely used in pediatrics has been on the rise around the world (Samanci and Pınarbaşı, 2023). These are the preferred antibiotics and are frequently used among outpatients and inpatients and is an important contributor to the development of resistance (Yüksel et al., 2006). We found that resistance of *E. coli* to the third-generation cephalosporin ceftriaxone was 58.9% and was higher than reported in other studies; 36.8% (Gul et al., 2022), 40.5% (Samanci and Pınarbaşı, 2023) and 36.7% (Cag et al., 2021). A study conducted in the United States utilizing 3511 *E. coli* urine isolates from 2014–2023 indicated showed high susceptibility to carbapenems (99.9–100%) (Mahajan et al., 2024), another study reported over 94% susceptibility to carbapenems (Isac et al., 2024). These levels were comparable to what we found (96.8%).

Although *E. coli* is highly sensitive to the broad-spectrum carbapenem drug meropenem, it should not be the first choice for empirical treatment (Samanci and Pınarbaşı, 2023). Nitrofurantoin is proposed as an excellent substitute for empiric antibiotic therapy in Chinese children with UTIs, especially since there is also an oral suspension form that has a good safety profile (Kara and Gurgoze, 2019; Mahdizade Ari et al., 2023; Squadrito and del Portal, 2025). In our study, resistance of *E. coli* to nitrofurantoin was low at 2.0% although comparable to that of other studies at 3.7% (Samanci and Pınarbaşı, 2023) and 4% (Păcurar et al., 2025). Therefore, nitrofurantoin is the only oral antibiotic with resistance levels < 5% that we can use in children.

Fluoroquinolone resistance levels in the current study for *E. coli*, *K. pneumoniae, E. faecalis, and E. faecium* were 38.4, 21.9, 19.9 and 63.7%, respectively and these levels were greater than the global rate of 20% reported by the Global Antimicrobial Resistance and Use Surveillance System (GLASS) (World Health Organization (WHO), 2022). EAU guidelines recommend fluoroquinolone and cephalosporins for empirical treatment of uncomplicated UTIs but suggest that fluoroquinolone resistance should be <10% to minimize treatment failure and resistance (EAU Guidelines, 2024). Our research indicated that fluoroquinolone is no longer suitable for the treatment of UTIs in children in the Chongqing area. The differences in the prevalence of antibiotic resistance in the above-mentioned studies might be due to the influence of the level of social and economic development and the prevalence of antibiotic use in various countries on the resistance patterns.

The resistance of *E. coli* and *K. pneumoniae* to antibacterial drugs also displayed differing trends from 2019 to 2024. We found that resistance of *E. coli* to amikacin decreased over time and this was contrary to the previously reported amikacin resistance trend (Samanci and Pınarbaşı, 2023). Resistance of *E. coli* to ciprofloxacin displayed a significant increasing trend (36.5% in 2019 vs. 50.2% in 2024), consistent with the trend of drug resistance in children reported previously in Bahrain (9.52% increased to 13.64%) (Shaaban et al., 2021). Our results indicated that yearly ampicillin/sulbactam resistance levels over a 6-year period for *E. coli* infections had been significantly reduced. The detection of carbapenem resistant-*E.coli* isolates from UTI patients reached its peak in 2021(5.8%) and this type of resistance was on the rise. The average resistance of *E. coli* to piperacillin/tazobactam and ceftriaxone were 5.5% and 59.0% respectively, showing an upward trend, while resistance to amikacin, gentamicin and nitrofurantoin (1.8, 34.2 and 2.0% respectively) displayed a downward trend. Studies from other countries reported different patterns i.e., resistance to amikacin, nitrofurantoin, imipenem and meropenem were stable (Kawalec et al., 2023; Abdelgalil et al., 2024; Mahajan et al., 2024; Bagnasco et al., 2025). In our study, detection of carbapenem resistant-*K. pneumoniae* isolates from UTI patients reached its peak in 2019(21.3%) this and was accompanied by high resistance levels of *K. pneumoniae* to ceftriaxone (50.4%), ampicillin/sulbactam (56.0%) and gentamicin(19.9%). In contrast, piperacillin/tazobactam (17.3% to 27.2%) and cefepime (27.7% to 44.6%) resistance levels increased.

Species of *Enterococcus* have also developed resistance to commonly used UTI antibiotics (Chen et al., 2020) and we confirmed that the resistance of *E. faecalis* and *E. faecium* to ciprofloxacin were 19.9% and 63.7%, respectively, greater than those levels reported in foreign studies (4.8% and 55.6%) (Cag et al., 2021). Furthermore, we did not identify *E. faecalis* isolates resistant to vancomycin and linezolid and the linezolid-resistance rate in our cohort was lower than the 4.8% reported in the literature (Cag et al., 2021), suggesting that vancomycin and linezolid remain effective against *E. faecalis* isolated from children with UTI.Although the resistance of *E. faecali*s to penicillin and nitrofurantoin was low (6.5% and 3.5%, respectively), it was observed that *E. faecium* was much more resistant to these antibiotics (91.5% and 29.7%, respectively, p<0.0001). However, the resistance of *E. faecalis* to high-level streptomycin was greater than that of *E. faecium*. In contrast, *E. faecalis* possessing vancomycin MICs ≥2 µg/mL (17.7%) was significantly higher than that of *E. faecium* (5.0%), and the proportion of *E. faecium* and *E. faecalis* isolates that possessed vancomycin MICs ≥2 µg/mL was not appreciably altered. *E. faecalis* also possessed linezolid MICs ≥ 2 µg/mL (69.0%) was significantly lower than that of *E. faecium* (82.0%) (P<0.05). At the same time, the proportion of *E. faecium* and *E. faecalis* isolates that possessed linezolid MICs ≥ 2 µg/mL displayed a significant downward trend (P<0.001). Therefore, the MICs of vancomycin did not drift, but the MICs of linezolid drifted in *E. faecium* and *E. faecalis*. The decreased MICs of E. faecalis to linezolid was more obvious and the trend began to emerge in 2022.

ESBL-producing *K. pneumoniae* comprised 54.2% of all isolated and this level was greater than the 16.0% reported previously (Bozo et al., 2025). Additionally, 58.6% of our isolates were β-lactamase -producing *E. coli* and these levels were greater than previously reported values of 19% (Păcurar et al., 2025) and 33.9% (Cag et al., 2021). Further analysis revealed obvious drug resistance patterns between ESBL-producing and non-ESBL-producing Enterobacteriales. In non-ESBL-producing *E. coli*, third and fourth-generation cephalosporins maintained excellent efficacy. In contrast, resistance of ESBL-producing *E. coli.* and *K. pneumoniae* to all the tested antibacterial drugs were all greater than non-ESBL-producing isolates and these results differed from previous studies (Ding et al., 2021; Shaaban et al., 2021; Bozo et al., 2025). Our research results confirmed that the sensitivity of ESBL-producing *E.coli* to imipenem and meropenem at 94.4 and 94.2% respectively, that were either lower or comparable to the 100% (Abdelgalil et al., 2024) and 93.6 and 93.6% (Păcurar et al., 2025) reported previously. Thus, efficacy has been retained. Therefore, in our study the response to carbapenems was consistent with their established role as last-line agents in ESBL-positive infections (Radu et al., 2024; Youseef et al., 2024; Abuelshayeb et al., 2025). Sensitivity of ESBL-producing *E.coli* to gentamicin was 60.9% and was lower than that a previous study (Abdelgalil et al., 2024), while we found nitrofurantoin and amikacin sensitivity were 96.1 and 97.2% and comparable to the 92 and 98% reported previously (Abdelgalil et al., 2024). Thus, amikacin remained effective in the majority of cases whereas gentamicin had lower activity. Ampicillin/sulbactam showed suboptimal efficacy for both groups and especially in ESBL-producing *E. coli* where resistance reached a combined level of 49.3%. This combination is therefore not recommended for use. These findings emphasize the urgent need to re-evaluate empirical treatment, especially in areas where a high prevalence of ESBL-producing *E. coli* is known.

In pediatric urinary tract infections, distinct features exist between pyelonephritis (upper-tract UTI) and cystitis (lower-tract UTI) in terms of virulence and pathogen distribution considerations. *E. coli* remains the predominant pathogen in both clinical entities, yet isolates causing pyelonephritis frequently express higher levels of virulence factors (e.g., P-fimbriae) facilitating renal parenchymal invasion (Daniel et al., 2023). Non-*E. coli* pathogens including *K.pneumoniae, P. aeruginosa* and enterococci tend to be more frequently recovered from children with pyelonephritis, especially among those with underlying congenital urinary tract anomalies (Zhan et al., 2025). Higher rates of ESBL-producing and multidrug-resistant isolates have been reported in upper-tract infections in several cohorts, although regional variations exist and some studies found no significant inter-group difference (Kılıç and Küçükkelepçe, 2025). Our age-stratified analysis further illustrates how these clinical and microbiological features converge. Consistent with established developmental patterns, male predominance in early infancy gives way to female predominance after the toilet-training period, and the relative proportions of *K. pneumoniae* and enterococci shift across age strata (≤1 year, 2–3 years, 4–6 years, ≥7 years). Our results showed rising annual resistance rates to nitrofurantoin and fluoroquinolones among *E. coli* and enterococci, which is consistent with previous surveillance data (Shkalim Zemer et al., 2024; You et al., 2026).The choice of antimicrobial agents requires particular caution in the pediatric setting. Nitrofurantoin achieves high urinary but poor renal-tissue concentrations; it is therefore appropriate for uncomplicated cystitis but not recommended for empirical treatment of pyelonephritis, in which tissue penetration is essential (Mahdizade Ari et al., 2023). Similarly, fluoroquinolones are restricted in children because of concerns regarding cartilage and tendon toxicity and are not recommended as first-line empirical agents for either form of pediatric UTI according to current guidelines, being reserved for selected cases with limited therapeutic alternatives (Ramírez et al., 2022).

Another finding of our study is that COVID-19 has changed the pathogen composition and antibiotic resistance pattern of paediatric UTIs. In post-COVID-19 pandemic, detection of *E.coli*, *E. faecium* and *K. pneumoniae* all decreased. This was especially true for *E.coli* where levels dropped from 45.3% (pre-COVID) to 35.0% (post-COVID). Consistent with a previous report(80.2 vs. 61.8%, respectively) (Gul et al., 2022), a possible reason may be that more hygienic lifestyle was followed post-COVID. Interestingly, for imipenem and meropenem, *E. coli* and *K. pneumoniae* had contrary levels of resistance; levels were highest during COVID and decreased post-COVID in *E. coli*, In contrast, *K. pneumoniae* resistance was lowest during COVID and increased post-COVID. Resistance of *E.coli* and *K. pneumoniae* to ampicillin/sulbactam, meropenem and gentamicin also displayed a decreasing trend post-COVID. The primary reason for this was most likely that the use of antibiotics declined during COVID. Antibiotic consumption and healthcare-seeking behavior declined markedly during the COVID-19 pandemic, potentially contributing to altered resistance dynamics in the pre-pandemic, pandemic and post-pandemic periods (Liang et al., 2024).our findings should therefore be interpreted in light of these shifting prescribing patterns.

### Limitations

This study has two limitations. The first one is a multicenter research in Chongqing area, which may limit the promotion of the results in other regions of China or countries with different antibiotic resistance profiles. In addition, the mechanism of resistant isolates could not be confirmed by molecular analyses due to the retrospective nature of the study.

## Conclusions

Our study revealed that significant shifts in bacterial profiles among pathogenic bacteria causing pediatric UTIs in the periods pre-, during and post-COVID-19 pandemic. Our data demonstrated that the detection of *E. coli*, *K. pneumoniae* and *E. faecium* all decreased after the epidemic. There was a significant reduction of carbapenem resistance-*K. pneumoniae*, coupled with rising of ESBL-producing *E. coli.*

## Data Availability

The raw data supporting the conclusions of this article will be made available by the authors, without undue reservation.
